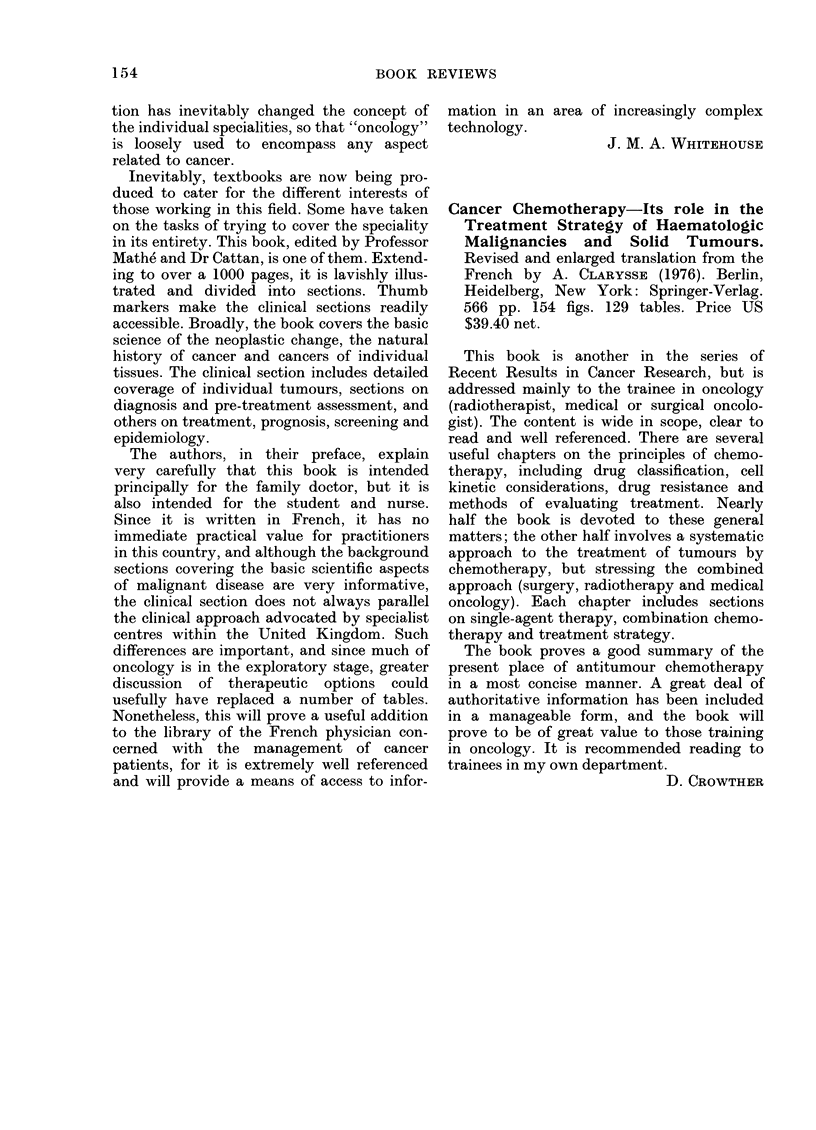# Cancer Chemotherapy—Its role in the Treatment Strategy of Haematologic Malignancies and Solid Tumours

**Published:** 1977-07

**Authors:** D. Crowther


**Cancer Chemotherapy—Its role in the Treatment Strategy of Haematologic Malignancies and Solid Tumours.** Revised and enlarged translation from the French by A. Clarysse (1976). Berlin, Heidelberg, New York: Springer-Verlag. 566 pp. 154 figs. 129 tables. Price US $39.40 net.

This book is another in the series of Recent Results in Cancer Research, but is addressed mainly to the trainee in oncology (radiotherapist, medical or surgical oncologist). The content is wide in scope, clear to read and well referenced. There are several useful chapters on the principles of chemotherapy, including drug classification, cell kinetic considerations, drug resistance and methods of evaluating treatment. Nearly half the book is devoted to these general matters; the other half involves a systematic approach to the treatment of tumours by chemotherapy, but stressing the combined approach (surgery, radiotherapy and medical oncology). Each chapter includes sections on single-agent therapy, combination chemotherapy and treatment strategy.

The book proves a good summary of the present place of antitumour chemotherapy in a most concise manner. A great deal of authoritative information has been included in a manageable form, and the book will prove to be of great value to those training in oncology. It is recommended reading to trainees in my own department.

D. Crowther